# Five Futures for Academic Medicine

**DOI:** 10.1371/journal.pmed.0020207

**Published:** 2005-07-05

**Authors:** Shally Awasthi, Jil Beardmore, Jocalyn Clark, Philip Hadridge, Hardi Madani, Ana Marusic, Gretchen Purcell, Margaret Rhoads, Karen Sliwa-Hähnle, Richard Smith, Tessa Tan-Torres Edejer, Peter Tugwell, Tim Underwood, Robyn Ward on behalf of the International Campaign to Revitalise Academic Medicine

## Abstract

The International Campaign to Revitalise Academic Medicine (ICRAM) considered current global instabilities and future drivers of change, and then created five scenarios of how academic medicine might look in 2025.

## Introduction

“Academic medicine” might be defined as the capacity of the health-care system to think, study, research, discover, evaluate, innovate, teach, learn, and improve. As such, little could be more important—particularly as new discoveries in science offer tremendous opportunities and emergent diseases pose huge threats. Yet something is not right with academic medicine. Worse, the diagnosis is not entirely clear, although many august bodies have reported on the issue (Table 1), and the treatment is unknown. Moreover, these previous consultations have been based in the industrialised world, particularly the United Kingdom and the United States, and few have taken a global view.

**Table 1:**
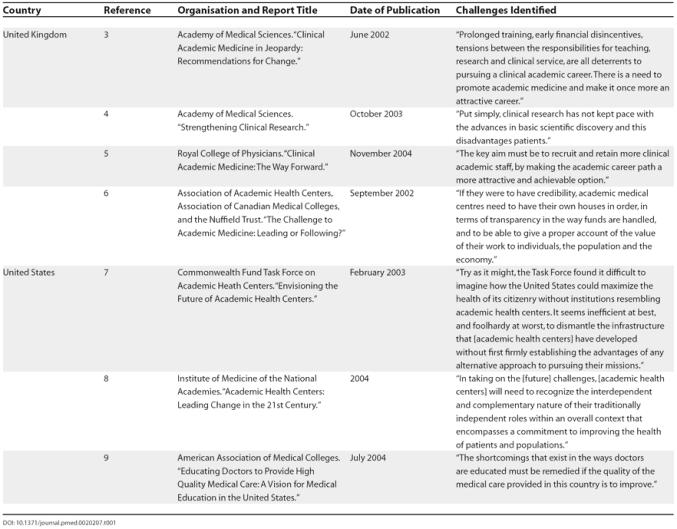
Reports of Major National Academic Medicine Organisations

The International Campaign to Revitalise Academic Medicine (ICRAM) was founded to give young medical academics an opportunity to think about the future of academic medicine (Box 1). It started with only two premises: (1) at the start of the new millennium it was necessary to think globally, and (2) “more of the same” was not the answer. Reinvention was needed.

**Box 1. ICRAM**

In 2003 the *BMJ*, *Lancet*, and 40 other partners launched ICRAM, a global initiative that is committed to fostering a debate about the future of academic medicine (bmj.com/academicmedicine). The campaign arose because of a persistent concern that academic medicine is in crisis around the world. At a time of increasing health burden, poverty, globalisation, and innovation, many have argued that academic medicine is nevertheless failing to realise its potential and global social responsibility.

ICRAM is composed of the following:

- A core working party of 20 medical academics representing 14 countries
- Stakeholder groups representing the areas of academia, business and industry, government and policy makers, journal editors, patients, professional associations, and students and trainees
- Regional groups covering the world
- A facilitating committee that helps plan and execute the ICRAM work

Through a series of stakeholder and regional consultations, systematic review of the available evidence, and future scenarios building, ICRAM intends to produce a series of recommendations for reform in global academic medicine, including the following:

- Developing a vision and values for academic medicine
- Recommending strategies for building capacity in academic medicine, including better career paths
- Proposing how academic medicine could improve its relationships with “customers,” including patients, policy makers, practitioners, and others

ICRAM decided to undertake some scenario planning, a technique of thinking about both the future and the present. The technique works by gathering together a team who consider the instabilities in the present and the drivers of the future and who then imagine plausible but different futures. The aim is not to predict the future, which is impossible, but to enable richer conversations through stretching thinking on what the future might bring. Once the scenarios have been created, they can be used to think more deeply about the present and the near future.

The group began by considering current instabilities in academic medicine around the world (Box 2). None of these ideas are new, and most people would probably agree with most of them.

**Box 2. Current Instabilities in Academic Medicine**

- Widespread, even universal, agreement that things are not right but little agreement on the exact nature of the problem
- Lack of capacity in “translational research”—that which brings innovations directly to patients
- The substantial gap between best, evidence-based practice and what actually happens
- The canyon between academics and practitioners
- The growing difficulty/impossibility of a single individual being competent in practice, research, and teaching
- Use of citation indices in research assessment, which overemphasises the value of basic research and underemphasises the importance of applied research that may bring more immediate benefit to patients
- A lack of mutual respect among different categories of researchers—basic, clinical, public health, primary care, applied, etc.
- Problems with career progression for academics
- Shortages of doctors wanting to enter careers in research
- In many, probably most, countries those doctors who enter careers in research, who ideally would be the “best and the brightest,” being likely to earn much less than those who can spend at least some time working in private practice
- Research often not being concerned with the biggest health problems (particularly true in a global context)
- Systems of accreditation of doctors making life difficult for doctors who want to pursue an academic career
- Although no clinicians are openly against research, clinicians being often unimpressed with doctors who concentrate on research
- Too much medical research being undertaken by doctors with limited training in research methods—making the research of poor quality
- Researchers often setting little store by quality improvement projects, although such projects are one of the ways of ensuring that all patients receive the most up-to-date care
- Much of the teaching in medicine being done by people with very little training in medicine
- The ratio of teachers to students in many medical schools being so low that the quality of teaching is reduced
- In many countries academic medicine lacking a well-resourced institution to speak for it
- Academic medicine relating poorly to its stakeholders—patients, policy makers, practitioners, the public, and the media
- The great pressures on health services, such that academic medicine is often squeezed and forgotten

Much of what determines the future of academic medicine will lie outside the control of medical academics themselves. The world will change around them, and they will have to follow. But there will also be change that comes from within academic medicine. Box 3 shows some of the drivers of the future considered by the group.

**Box 3. Drivers of Change in Academic Medicine**

- New science and technology, particularly genetics and information technology
- The rise of sophisticated consumers
- Globalisation
- Emergent diseases
- Increasing gap between rich and poor
- Death of distance
- “Big hungry buyers” demanding more from health care
- Spread of the internet and digitalisation
- Managerialism
- Increasing anxieties about security
- Expanding gap between what could be done and what can be afforded in health care
- Lack of agreement on where “health” begins and ends
- Ageing of society
- Feminisation of medicine
- Increasing accountability of all institutions
- Loss of respect for experts
- Rise of self care
- Rise of ethical issues
- 24/7 society
- Economic and political rise of India and China

## Five Scenarios

In building scenarios, the group used a time span of 20 years, but some of the scenarios are more futuristic than others. We decided to write up the scenarios as if they were in the past in order to give some idea of how they might arise. The scenarios are summarised in Table 2, and described more fully in the rest of this article.

**Table 2:**
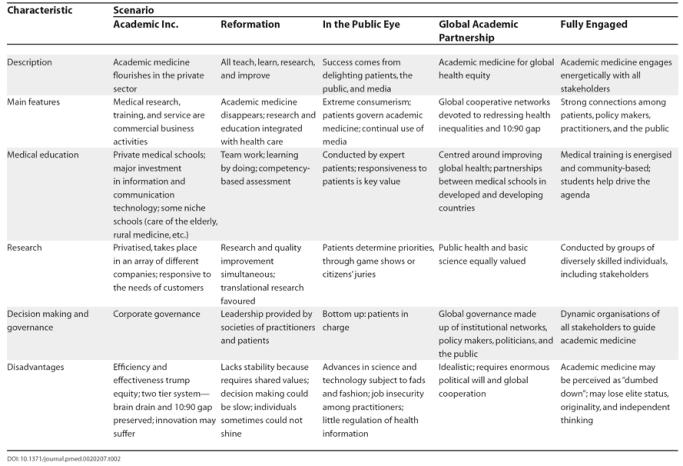
Summary of Scenarios

**Academic Inc.: “Academic medicine flourishes in the private sector”.** Slowly but surely the public sector around the world realised that it could not support the costs of academic medicine. Medical students had high earnings during a professional lifetime: why shouldn't they pay for their education? And if researchers were doing something valuable then shouldn't they be able to find a market for their product—accepting that sometimes payment would come from the public sector?

The process of academic medicine moving almost entirely into the private sector began with an increasing number of medical schools becoming private. The most prestigious schools went first. In an increasingly global market these schools could charge high fees, pay their staff well, and improve their facilities. They also invested a great deal in information and communication technology, bringing state-of-the-art learning to their students. This meant too that the schools could run courses for students far away from their geographical base. As these schools developed they expanded internationally, sometimes forming alliances with other prestigious schools but also taking over weaker schools. Soon the best schools were operating on all five continents. In the branches in developing countries, medical student bodies tended to comprise both students from the developed countries along with a quota from the developing countries.

Competition was intense and involved both cost and quality. Schools that managed to improve quality while reducing costs—usually through clever use of technology—flourished, but a great many medical schools disappeared. The numbers of students, however, increased, and the competition for talent was intense, with schools offering generous bursaries to poor but bright students and becoming ever more sophisticated at finding high-quality students in resource-poor populations.

As happens in most intensely competitive markets, medical schools also competed by occupying niches. Schools offered very different kinds of courses, specialising in older students, basic science, rural medicine, surgical skills, training doctors for poor communities (in both the country where the medical school was based and lower and middle income countries), and many other subject areas. Sometimes the students' fees were paid by governments, local communities, or the military in order to produce students who met their needs. Many students attended schools in countries other than their own.

Health research happened almost entirely in the private sector, but in a wide range of organisations: pharmaceutical companies, medical schools, biotechnology companies, small companies offering a huge range of services, and charities. Companies were founded not only by researchers but also by patients, practitioners, and others. Many of the companies founded by academics offered complex and innovative heath services. As in all business, to be successful companies had to be highly responsive to the needs of customers, including patients and governments. Those that were innovative, flexible, responsive, and relentlessly cost-conscious flourished, but many companies “failed”. Little stigma was, however, attached to “failure”. Indeed, as in Silicon Valley at the end of the 20th century, experience of “failure” was seen by many as an important qualification in a leader.

The injection of more competitive pressure and a competitive business model into academic medicine increased not only efficiency but also “effectiveness”: research was much more relevant, and the time lag between the development of new ideas and their introduction into practice was dramatically shortened. Basic science was still well funded because both governments and investors recognised the potentially high returns. Research into the health needs of poor and marginal populations also improved because public sector bodies concentrated their resources into these problems, leaving the problems of the wealthier to the market.

Applying a competitive business model to academic medicine meant that efficiency and effectiveness trumped equity. On the negative side, the scenario “Academic Inc.” resulted in a two-tier system, with the rich finding it easy to create careers in academic medicine and the poor finding it hard to enter the profession—despite the generous bursaries available to some. In addition, much more attention continued to be paid to the health problems of wealthier people and countries, and the brain drain from poor to rich countries accelerated. Innovation also suffered. Private academic medicine enjoyed less lead time and had more direct and immediate accountability to its shareholders than when it was publicly funded.

**Reformation: “All teach, learn, research, and improve”.** Twenty years ago there was increasing concern about the gap between academic medicine and practice, with important research results not being implemented, too much irrelevant research, bored students, and practitioners who stopped learning. In some medical communities and among their medical leadership, the response was not to try and strengthen academic medicine and make it more responsive but rather to abolish it and instead to bring the processes of teaching, learning, researching, and improving into the main stream of health care, and tailor these to local needs. This innovative—though not initially welcomed—response proved to be highly successful and was copied everywhere. A century of separation of academic medicine from practice was ended. Professors disappeared. The entity “academic medicine” was dead. It was akin to the destruction of the monasteries and so became known as the reformation of academic medicine.

Teaching, learning, researching, and quality improvement all began to take place in the practice setting and were everybody's business. But importantly the fiction that an individual could be competent in practice, teaching, research, and improvement finished. It was teams that had to have all these competencies not individuals, and substantial investment was made in getting teams to work well and to communicate to a degree rarely seen before in health care.

The teams were supported by advanced technology that provided online learning, decision support, answers to questions that arose during practice, and access to research results. Much of the teaching and research was done in collaboration with patients, and all teams included patients as well as practitioners, students, professional researchers, and other health professionals.

Research was built around the questions that arose when doctors (and other health professionals) and patients consulted together. The questions were collected by the National Question Answering Service, which provided evidence-based answers to questions when it could. The service also organised research to answer questions that arose commonly, were unanswered at that time, but could be answered. Teams of different sizes and skills were assembled to conduct research; some of the researchers were permanently in practice but others—particularly basic researchers—were resident in research institutions and joined teams as needed.

Some research was driven by discoveries made in basic science rather than questions that arose during practice; the fact that practitioners and researchers were used to working together in teams facilitated “translational” research.

The teams also switched back and forth from research to quality improvement, ensuring that research developments were fed through into practice. Studies reporting the results of quality improvement projects were published just as frequently as research studies, and highly efficient information systems ensured that relevant information reached practitioners quickly—unlike in the old days when practitioners had been deluged with research results, most of which were irrelevant to them.

Intellectual leadership in health care was provided by specialist societies, which included patients, citizen juries, researchers, health professionals other than doctors, health managers, and policy makers as equal members. In most countries the specialist societies were gathered together into an academy or institute of health care, and there was an international academy that was much more than a talking shop: it had a strong influence on world leaders.

Health students started their training with six months in institutions that taught them how to learn. The learning professionals who staffed these institutions were available to all the practice teams. Students then learnt through attachment to practice teams, starting with a spell in general practice. Some students specialised early, some becoming competent cardiologists within five years. Becoming an independent professional depended not on university degrees or exams but on a demonstration of competencies determined by a national body dominated by patients that used the most up-to-date methods for assessment.

Learning was by doing, and the divisions of undergraduate, specialist, and continuing education disappeared—as did the divisions among teaching, learning, researching, and quality improvement.

One problem with the “Reformation” scenario was that it lacked stability because it required shared values and beliefs, which not all teams held. Plus decision making could be slow, and it was hard for brilliant and charismatic individuals to shine as leaders and thinkers. One result was that such individuals eschewed careers in health care, research, and education. In developing countries, in particular, the lack of an academic medicine structure meant that there were fewer opportunities to influence medical research and training.

**In the public eye: “Success comes from delighting patients and the public”.** Academic medicine was slow to recognise the rise of global media, “celebrity culture,” and the use of public relations (or spin) to drive the political process, but once it did recognise how the world had changed it responded dramatically. Whereas it had been suspicious of the media and public appeal and rather patronising to patients, academic medicine realised that to succeed it must delight patients and the public and learn to use the media. The most successful academics became those who were very responsive to patients and the public, capturing their imaginations, and appearing regularly on their television screens. Some medical academics became as well known as film and rock stars and were feted by politicians.

All academic institutions became dominated by public citizens and patients, and the public and media relations department became the most important department in any institution. Money—from both public and private sources—followed “interest,” which was often influenced through game and “reality” shows on television. Academic medicine learnt from sport, and large prizes were awarded to those who won academic competitions. Although some academics were horrified by these developments, others remembered how John Harrison had been stimulated to solve the problem of calculating longitude through the promise of a large prize.

Not all decisions on research priorities and resource allocation were made in the glare of television cameras. Although all decisions put the public interest first and were made by the representatives of the public, some were made by more sedate and evidence-driven bodies like citizens' juries, where randomly selected patients and members of the public were presented with detailed evidence by “experts.”

Medical training was also conducted in the public eye, with students receiving much of their training from expert patients. The agenda for training was set predominantly by the public and patients, and responsiveness to patients was the number one characteristic of successful doctors and students.

There was much greater diversity than at the beginning of the millennium in the form and size of academic institutions, with both huge public and private universities and smaller institutions that were often built around one charismatic individual. Competition among the institutions was intense—particularly for “celebrity” teachers and researchers. Only those institutions that could attract and keep public attention could survive.

In developing countries, the academic health community linked itself with strong consumer movements, such as those focused on HIV/AIDS, and the leading nongovernmental organisations established their own medical schools. These ensured a powerful public voice, that training was tailored to local need, and a committed group for field testing new research advances.

On the negative side in the “In the Public Eye” scenario, medical academics felt more anxious about their job security and ability to succeed. Even celebrity academics worried their time in the spotlight was short lived. Advances in science, medicine, and technology were shaped by popular appeal, and thus subject to fads and fashion. Some patients struggled with their new-found status as governors, and there was little regulation of health information.

**Global academic partnership: “Academic medicine for global health equity”.** In 2005, the world began to find the growing global gap between the rich and poor unacceptable. The concern was driven partly by the media and global travel bringing the plight of the poor in front of the eyes of the rich, but it was also driven by anxieties over global security. Terrorism was recognised to be fuelled by the gap between rich and poor. Global policy makers also understood better—particularly after the report of the Commission on Macroeconomics and Health [2]—that investment in health produced some of the richest returns in not only social but also economic development. Health care was a “must have” not a “nice to have”.

Money flowed into health in the poor world, and governments required that the investment be accompanied by learning, research, planning, and evaluation. The primary concern of much of academic medicine became to improve global health, particularly through concentrating on the health problems of the 90% who had previously received only 10% of health care resources (the 90:10 gap). Academics became excited by this kind of work not only because it was intellectually exciting and highly rewarding in personal terms but also because it was where money and prestige were most likely to be found. The result was that it was impossible for an academic institution to be a world leader without a substantial investment in global health and extensive links around the world.

The view and scope of academic medicine broadened. It was increasingly concerned with human rights, justice, economics, and the environment, recognising that these are the major drivers of health. This broader view meant that academic medicine (usually referred to as “global health innovation” by 2012) became the main institution concerned with the rights of those who will be alive 50 years from now, a group that previously had nobody to speak for them. But at the same time basic science remained important because of the contribution it could make to global problems like finding vaccines and new treatments for malaria, AIDS, and emergent diseases like rapidly spread respiratory virus, which appeared in 2010 and killed millions in a global pandemic.

Academic medicine, in partnership with governments (and where corruption is prevalent, with nongovernmental organisations), became a major driver towards achieving the millennium development goals. The G8 governments had signed an accord that prohibited recruitment of academic health professionals from developing countries. Medical schools and research institutions formed themselves into networks linking with local nongovernmental organisations, joining developed and developing countries and forming links among developing countries. A network was formed whereby the universities in developed countries committed 10% of their faculty members' time to addressing problems of the developing world. Some institutions formed developed country–developing country pairs, some merged, and researchers, teachers, and students moved regularly between both settings. The net flow was to the developing world, with the 90:10 divide beginning to correct itself surprisingly rapidly. Big investments in information and communication technology meant that those in developing countries had the same access to information and modern learning methods as those in developed countries.

The networks of institutions developed a global governance structure with substantial input from politicians, practitioners, policy makers, the public, and patients. Academic medicine moved from being marginal to central in global affairs, and medical academics, particularly those with experience in both the developing and developed world, became global leaders. It was a development that happened naturally because of their broad interests in human rights, justice, and the environment.

The “Global Academic Partnership” scenario for academic medicine, however, was idealistic and sometimes struggled with realising its full potential—despite the best intentions of its architects and practitioners—because it required enormous political will and global cooperation. Too often nations would revert to narrow self interest. Academics as well often longed for the comforts of the developed world and sometimes felt exhausted by extensive travelling and the enormous problems of the developing world.

**Fully engaged: “Academic medicine engages energetically with all stakeholders”.** Early in the new millennium academic medicine became concerned that its relationships with its stakeholders were mostly poor. The public had little or no understanding of what academic medicine was or why it mattered. Its very name implied irrelevance to many. Patients often felt patronised by academics, and many practitioners—including doctors—were unconvinced of the value of academic medicine. “I wouldn't want a professor of surgery touching me,” was a commonly heard refrain. Although some leading academics did have good relationships with politicians, policy makers found that many academics were not interested in policy problems and that the studies they produced, even if relevant, came too late to be useful. Policy makers recognised that biotechnology might be very important in future wealth creation, but it was difficult to fund because the public profile of academic medicine was both low and clouded.

Most medical academics recognised that they were doing a poor job of relating to stakeholders and that it was thus unsurprising that they were misunderstood, underappreciated, and seen as largely irrelevant. This, they thought, was particularly unfortunate as the ability of the system of health care to discover, think, study, learn, and evaluate had never been more important.

The medical academic community thus decided that it had to do better, and across the globe medical academics organised themselves to engage fully with their stakeholders. In many countries this meant the creation of new organisations. In others it involved the transformation of existing organisations: the gongs and gowns were abandoned, and focus groups began. Fifty prestigious universities in developed countries with medical faculties partnered with universities in developing countries to help stop the “brain drain” and replace it with a “brain gain” through incentive programs that provided resources for training and research, academic recognition, travel funds, and family support. Everywhere medical academics had to learn how to communicate with the public, patients, and practitioners. They had to stop being elitist and patronising and recognise the messiness of public discourse. Crucially, they had to be much cleverer in handling the media, telling them not only about their successes but also sharing their uncertainties and problems.

But communication on its own wasn't enough. Academic medicine had to bring its stakeholders inside its processes. The governance of academies included patients, the public, practitioners, health administrators, and policy makers. Sometimes the president of an academy was not a distinguished researcher but a prominent patient, journalist, or community organisation leader. The medical academics discovered that their arguments were taken much more seriously when advanced clearly by a patient rather than by themselves. Patients, health administrators, and community organisation representatives became involved not only in peer review of grants and studies but also in the prioritising, designing, and conducting of research. Medical students became the main drivers of medical education rather than simply its consumers.

Slowly but surely medical academics became not a group apart but a highly diverse group of people with a broad set of skills and backgrounds. They were at the centre of a vibrant community of patients, members of the public, practitioners of all stripes, policy makers, members of the media, marketing experts, and politicians, all of whom were interested in learning, studying, researching, and thinking about health care.

Some academics, it must be said, found the change uncomfortable and were unconvinced of its value. Critics talked of “dumbing down” and popularisation. They fretted that in abandoning its elitism academic medicine had lost its ability to be truly original and speak independently.

For most, however, academic medicine was so much more fun than it used to be. Applications to medical schools increased. Health services invested more in evaluating what they did and paid more attention to the results. More funds flowed into basic research, and there were improvements in the connections between the many diverse groups involved in research—with the result that intellectual silos were breached.

## Lessons from the Scenarios

None of these scenarios will come to exist as we have described them, but the future is likely to contain some elements from each of them. We have tried to identify common features in the scenarios to learn lessons for now. These features are shown in Box 4. Our main hope for these scenarios is that other groups may find them useful in thinking about both the present and the future of academic medicine. The scenarios will need to be adapted to the particular social, economic, and political conditions of different regional and national settings. As such, they are tools that can be used globally, modified as required. We seek not agreement but broader thinking.

**Box 4. Common Features Shared By All Five Scenarios**

- It is likely to be important in all scenarios for academic medicine to put more effort into relating to its stakeholders: the public, patients, practitioners, politicians, and policy makers. This may demand the development of new institutions that involve all these groups.
- Academic institutions will need to be more globally minded.
- Teaching, researching, improving, leading, and providing service will continue to be important, but expecting individuals to be competent in them all will be increasingly impractical.
- Teamwork will become ever more important, but it will also be necessary to allow individuals to shine and flourish.
- Competition among academic institutions is likely to increase, and the competition will increasingly be international.
- Academic institutions will need to become more “business-like” in all the scenarios. They will also need to be more adept at using the media.
- Teaching and learning will be increasingly important—not least because dissatisfied students may go elsewhere. Learning will be lifelong and will depend heavily on information technology.
- It will be increasingly important to combine research, both basic and applied, with implementation and improvement. The gap between knowledge and practice will become increasingly intolerable.
- The range of types of academic institutions is likely to become increasingly diverse, with medical schools or academic centres just one form.
- Academic medicine will need to be ever broader in its thinking and skill set, combining with and learning from other disciplines such as economics, law, ecology, and humanities.
- Thinking about the future will become increasingly important for academic institutions but also increasingly difficult.

## Abbreviation

ICRAM: International Campaign to Revitalise Academic Medicine
